# Supplementary material for: A niche-derived nonribosomal peptide triggers planarian sexual development
Source: Proc Natl Acad Sci U S A. 2024 Jun 18;121(26):e2321349121. doi: 10.1073/pnas.2321349121 (PMC11214079; doi:10.1073/pnas.2321349121)
Supplement: Supplementary file 1 — Appendix 01 (PDF) [file pnas.2321349121.sapp.pdf]

Supporting Information for

A niche-derived nonribosomal peptide triggers planarian sexual development.

Melanie Issigonis, Katherine L. Browder, Rui Chen, James J. Collins III, Phillip A. Newmark

Phillip A. Newmark
Email: pnewmark@morgridge.org

This PDF file includes:

Figures S1 to S7
Tables S1 to S2
SI References

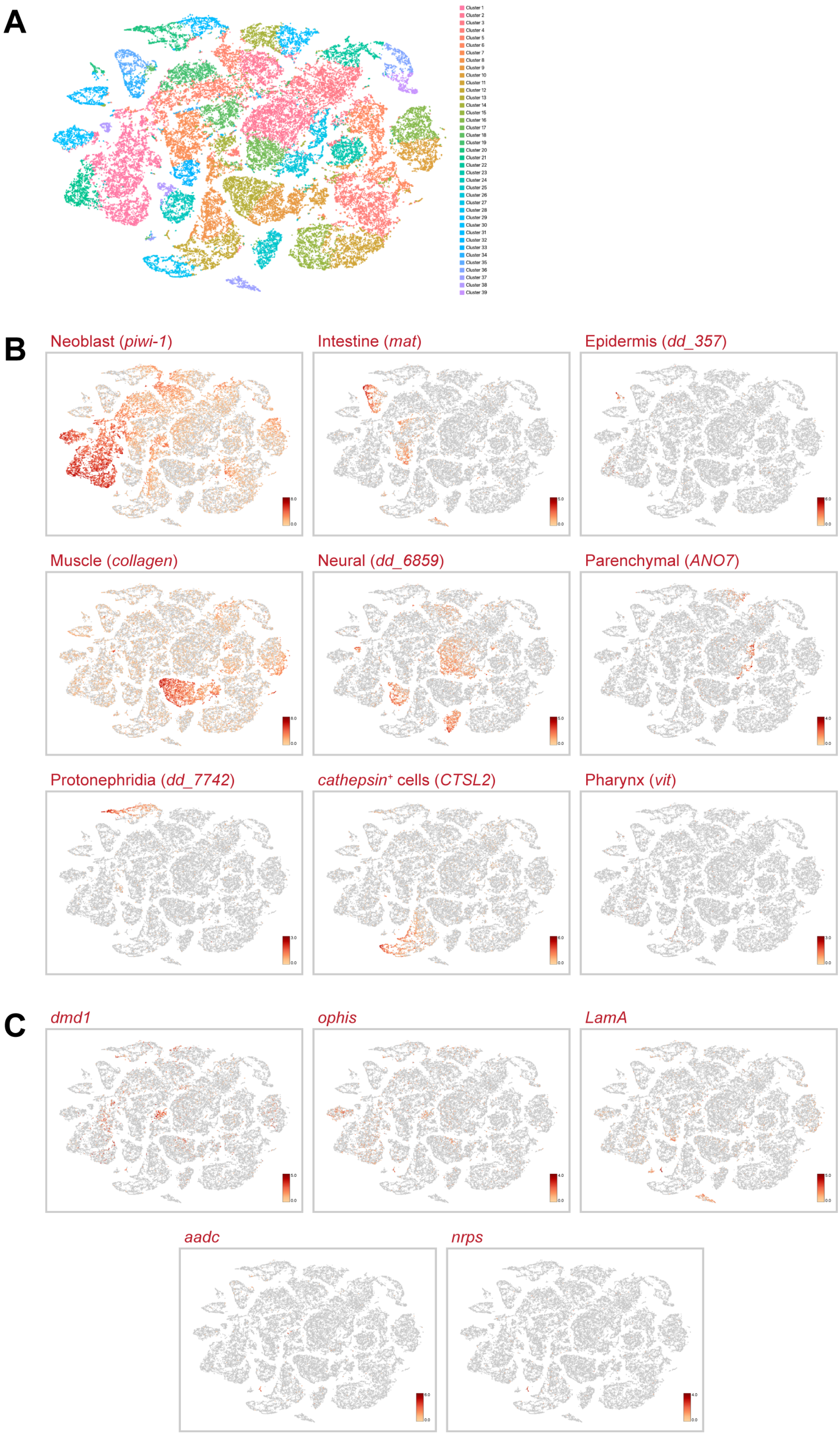
A
Cluster 1
Cluster 2
Cluster 3
Cluster 4
Cluster 5
Cluster 6
Cluster 7
Cluster 8
Cluster 9
Cluster 10
Cluster 11
Cluster 12
Cluster 13
Cluster 14
Cluster 15
Cluster 16
Cluster 17
Cluster 18
Cluster 19
Cluster 20
Cluster 21
Cluster 22
Cluster 23
Cluster 24
Cluster 25
Cluster 26
Cluster 27
Cluster 28
Cluster 29
Cluster 30
Cluster 31
Cluster 32
Cluster 33
Cluster 34
Cluster 35
Cluster 36
Cluster 37
Cluster 38
Cluster 39
B
Neoblast (*piwi-1*)
Intestine (*mat*)
Epidermis (*dd_357*)
Muscle (*collagen*)
Neural (*dd_6859*)
Parenchymal (*ANO7*)
Protonephridia (*dd_7742*)
cathepsin+ cells (*CTSL2*)
Pharynx (*vit*)
C
dmd1
ophis
LamA
aadc
nrps

Fig. S1. Single-cell RNA sequencing of cells from sexual *S. mediterranea*. (**A**) t-SNE plot of 39 clusters. **(B)** t-SNE plots of representative genes for nine major planarian tissue classes previously characterized in asexual *S. mediterranea* (1). All somatic tissue classes are present except for pharyngeal cells, which are not detected in this sexual scRNA-seq dataset since we enriched for reproductive tissues lacking this organ. (**C**) t-SNE plots showing expression of somatic gonadal gene markers *dmd1*, *ophis*, *LamA*, *aadc*, and *nrps*.

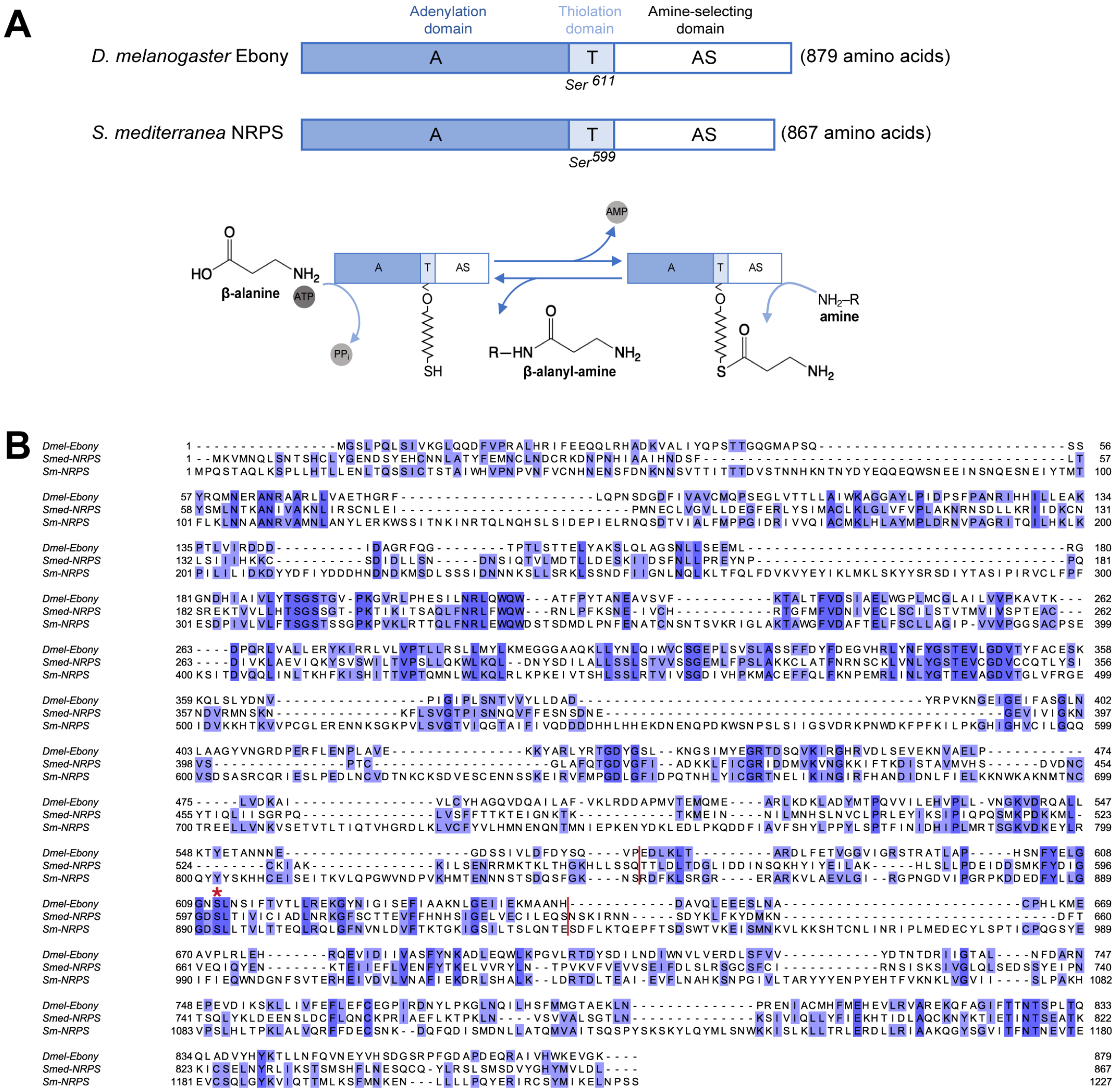

Fig. S2. *Smed-nrps* encodes a non-ribosomal peptide synthetase. (**A**) Schematic showing adenylation (A), thiolation (T), and amine-selecting (AS) domains in *D. melanogaster* Ebony and the *Schmidtea mediterranea* homolog NRPS. *Drosophila* and *S. mediterranea* share a conserved serine residue in their thiolation domains. NRPS proteins like Ebony can conjugate β-alanine to various biogenic amines (e.g., dopamine, histamine, etc.). This enzymatic process involves three steps: adenylation of β-alanine catalyzed by the adenylation (A) domain; covalent attachment of β-alanine to a phosphopantetheinyl group on a conserved serine within the thiolation (T) domain; and binding of an amine in the amine-selecting (AS) domain, which facilitates nucleophilic attack of the NRPS-bound β-alanine resulting in a β-alanyl-amine dipeptide product. (**B**) Protein alignment of *Drosophila* Ebony, *S. mediterranea* NRPS, and *S. mansoni* NRPS. The serine thiolation site (marked by an asterisk) in the T domain (delineated by red lines) is conserved.

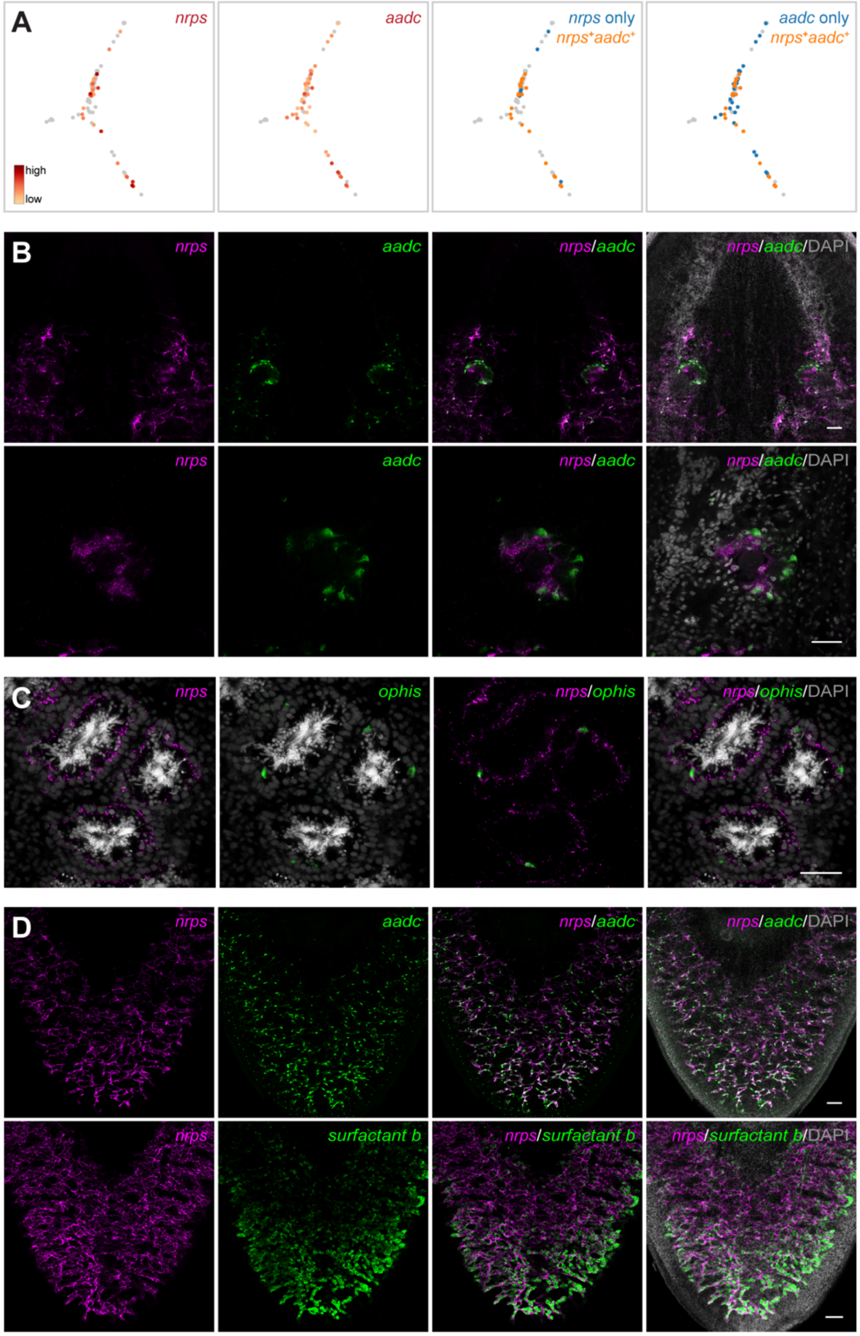

Fig. S3. *nrps* is expressed in somatic gonadal cells. (**A**) Cluster of 73 cells highly enriched for somatic gonadal transcripts contains 28 *nrps*$^+$ cells and 48 *aadc*$^+$ cells (red); 22 cells coexpress *nrps* and *aadc* (orange). (**B**) Projection of ventral head region (top) and confocal section of ovary (bottom) showing dFISH of *nrps* (magenta) and *aadc* (green). (**C**) Confocal section of testes with *nrps* (magenta; cytoplasmic localization) and *ophis* (green; nuclear) coexpressing cells. *ophis* RNA localizes mainly to the nucleus of somatic gonadal cells, which extend long *nrps*$^+$ cytoplasmic projections that encyst developing germ cells. (**D**) Projections of confocal sections showing dFISH of *nrps* (magenta) with *aadc* (green; top), or yolk cell marker *surfactant b* (green; bottom) in the ventral posterior region of sexually mature planarians. Nuclei are counterstained with DAPI (gray; **B-D**). Scale bars, 100 μm (**B**, top; **D**), 50 μm (**B**, bottom; **C**).

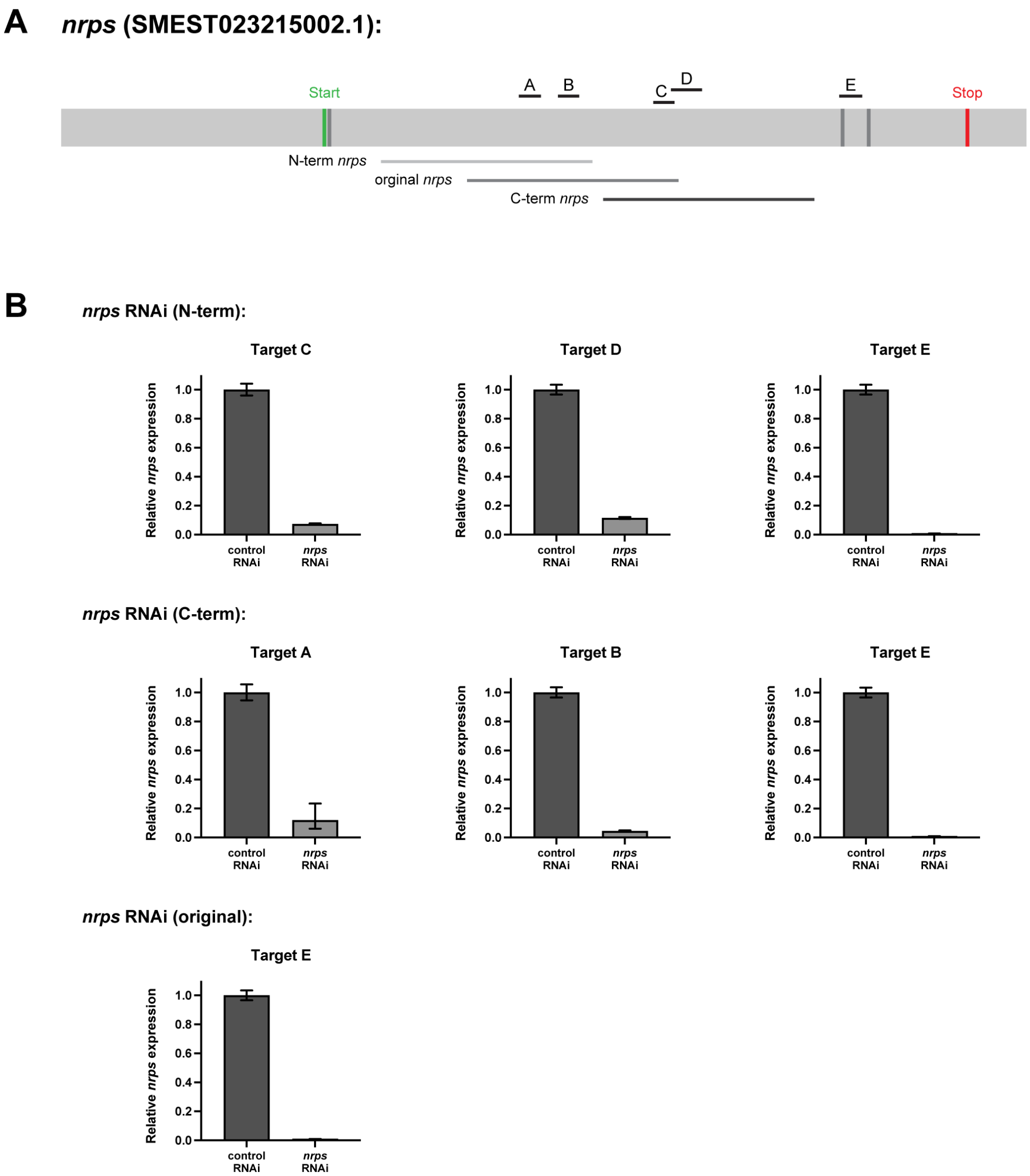

Fig. S4. Testing *nrps* RNAi specificity and quantifying *nrps* expression in knockdown animals. (**A**) To test for *nrps* RNAi specificity and exclude the possibility of off-target effects, RNAi was performed with dsRNA targeting three ~1 kb regions of *nrps*: the original region used throughout this study, and 2 non-overlapping regions (N-terminus vs C-terminus). *nrps* gene (gray bar) is shown with positions for start (green) and stop (red) codons, exon-exon boundaries (dark gray), cloned regions (bottom), and qPCR amplicons (top: A-E). (**B**) qPCR analysis of *nrps* mRNA expression normalized to *β-tubulin* in control and *nrps* RNAi animals depicting efficient knockdown of *nrps* after RNAi. Top: dsRNA targeting the N-terminus of *nrps* was used for RNAi-mediated knockdown of *nrps*, and qPCR primers targeting regions C, D, and E were used to quantify *nrps* expression levels. Middle: dsRNA targeting the C-terminus of *nrps* was used for RNAi and qPCR primers targeting regions A, B, and E were used to quantify *nrps* expression levels. Bottom: dsRNA targeting the original cloned amplicon of *nrps* and qPCR primers targeting region E were used to quantify *nrps* expression levels. N = 4 biological replicates (3 technical replicates each). Bar graphs depict relative quantification ($2^{-\Delta\Delta Ct}$) values normalized to control RNAi with 95% confidence intervals.

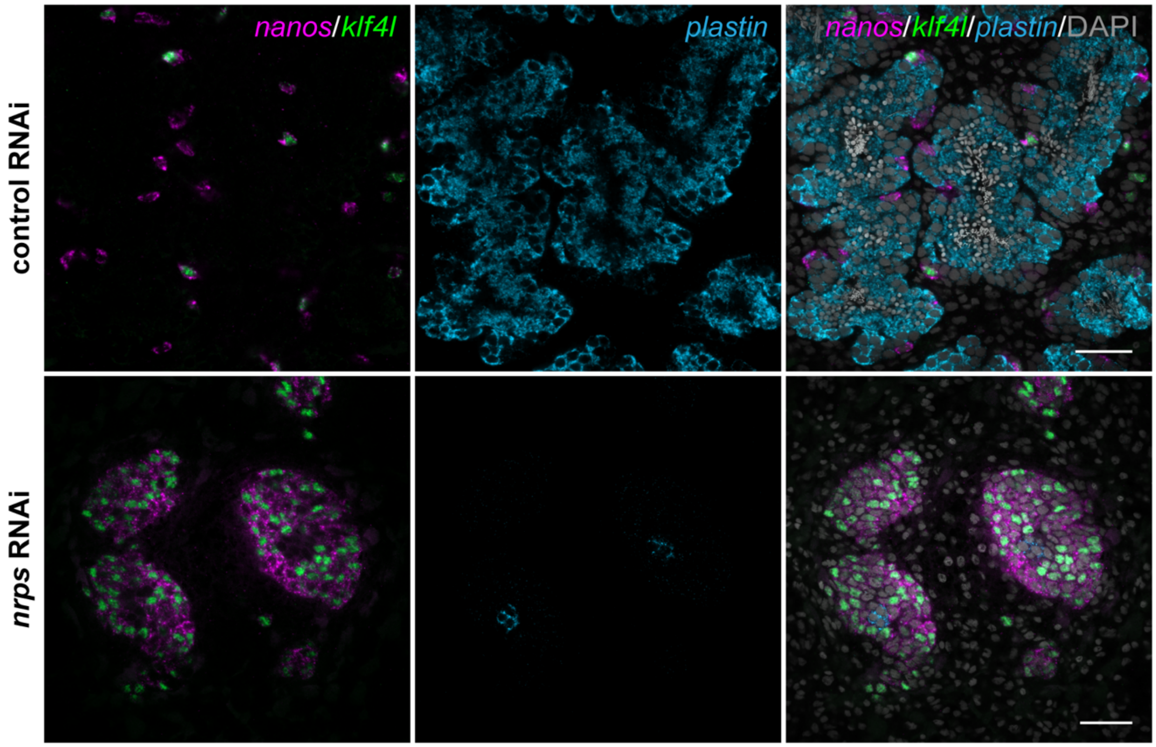

Fig. S5. Hyperplastic testes in *nrps* RNAi animals lack sperm and have few differentiating spermatogonia. dFISH of *klf4l* (green), *nanos* (magenta), and *plastin* (differentiating spermatogonia, spermatocytes, spermatids; cyan) in testes of sexually mature *nrps* RNAi planarian. *nrps* RNAi results in hyperplastic testes filled with *klf4l*$^{+}$ *nanos*$^{+}$ early germ cells. 41% of testes (249/614 testes; n=12 animals) contain very few differentiating *plastin*$^{+}$ spermatogonia. Nuclei are counterstained with DAPI (gray). Scale bar, 50 μm.

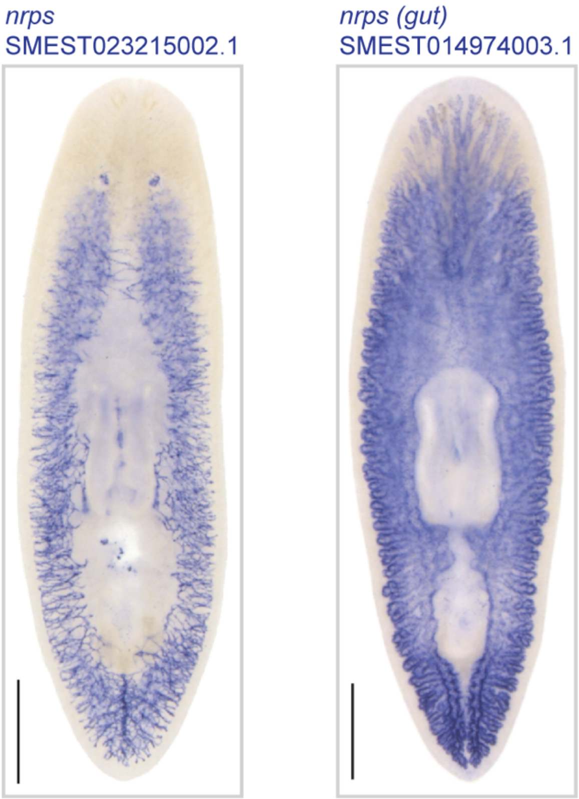

Fig. S6. Colorimetric in situ hybridization of *nrps* paralogs. *nrps* transcript is expressed in the reproductive system and a paralogous *nrps* is expressed in the gut in adult sexual *S. mediterranea*. Scale bars, 1 mm.

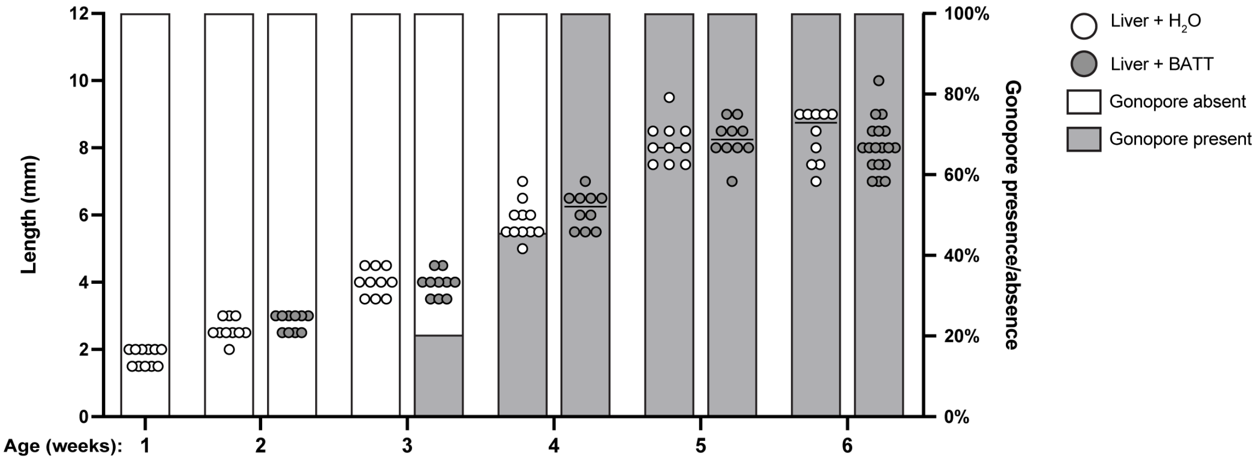

Fig. S7. BATT triggers sexual maturation. Quantification of sexual planarian length (mm; left Y axis; horizontal line represents median) and gonopore presence (right Y axis) during development. One-week old hatchlings were fed liver +/–BATT for 6 weeks. Supplementation with BATT did not affect growth but triggered precocious sexual maturation (evidenced by the presence of a gonopore) in +BATT individuals. n=10-18 planarians per time point.

Table S1. Information for transcripts mentioned in this paper.

Gene	Reference	Sequence information
nrps	This paper	Planmine SMEST.1: SMEST023215002.1
***nrps* (gut)**	This paper	Planmine SMEST.1: SMEST014974003.1
klf4l	(2)	Planmine SMEST.1: SMEST031008001.1
Laminin A	(2)	Planmine SMEST.1: SMEST056013009.1
delta3	(3)	Genbank accession: OL957299
nanos	(4)	Genbank accession: EF035555.1
dmd1	(5)	Genbank accession: KC736555.1
ophis	(6)	Genbank accession: KX018822.1
surfactant b	(7)	Genbank accession: KY847536.1
plastin	(8)	Genbank accession: DN311193.1
	(9)	Genbank accession: HO007660.1
pka	(8)	Genbank accession: DN316100.1
	(9)	Genbank accession: HO007035.1
tph	(10)	Genbank accession: KF134114.1
aadc	(10)	Genbank accession: KF134115.1

Table S2. Primer sequences.

**Cloning into pJC53.2**	**Forward**	**Reverse**
nrps	TCGTTTGCCACAGAACAGGA	CGATTAGGCCGTCGGTTAGG
***nrps* (gut)**	CCGATTTTGGCAGCTTCTGG	CTCAGCCACCCATTTCGTCT
***nrps* (N-term)**	TGGATGAAGGCTTTGAGAGG	GATTCGGCCGCAAATAAATA
***nrps* (C-term)**	ACAGCCGTTATGGTCCACTC	ACCGACACGTTTAGCTTTGG
**qPCR**	**Forward**	**Reverse**
β-tubulin	TGGCTGCTTGTGATCCAAGA	AAATTGCCGCAACAGTCAAATA
**Target A**	TCGTAAGCAGCGGTGAAAT	CTTCCGTTGAGCCGTAAAGA
**Target B**	GCACACCCATTTCGAACAATC	CAGTCCACAAGTCGGTGATAC
**Target C**	TGCTCTGTAAGATTGCGAAGA	GTCTAGAGTGGTTTGGGATGAG
**Target D**	CCCAAACCACTCTAGACCTAAC	GAGTCACCACCAATATCGTAGAA
**Target E**	ACGTCTGAAGCAACGAAGAA	GTGAGCGCAAATATTGACATTGA

SI References

1. C. T. Fincher, O. Wurtzel, T. de Hoog, K. M. Kravarik, P. W. Reddien, Cell type transcriptome atlas for the planarian *Schmidtea mediterranea*. *Science* **360**, eaaq1736 (2018).
2. M. Issigonis, *et al.*, A Krüppel-like factor is required for development and regeneration of germline and yolk cells from somatic stem cells in planarians. *PLOS Biol.* **20**, e3001472 (2022).
3. U. W. Khan, P. A. Newmark, Somatic regulation of female germ cell regeneration and development in planarians. *Cell Rep.* **38**, 110525 (2022).
4. Y. Wang, R. M. Zayas, T. Guo, P. A. Newmark, *nanos* function is essential for development and regeneration of planarian germ cells. *Proc. Natl. Acad. Sci. U.S.A.* **104**, 5901–5906 (2007).
5. T. Chong, J. J. Collins, J. L. Brubacher, D. Zarkower, P. A. Newmark, A sex-specific transcription factor controls male identity in a simultaneous hermaphrodite. *Nat. Commun.* **4**, 1814 (2013).
6. A. Saberi, A. Jamal, I. Beets, L. Schoofs, P. A. Newmark, GPCRs direct germline development and somatic gonad function in planarians. *PLOS Biol.* **14**, e1002457 (2016).
7. L. Rouhana, J. Tasaki, A. Saberi, P. A. Newmark, Genetic dissection of the planarian reproductive system through characterization of *Schmidtea mediterranea* CPEB homologs. *Dev. Biol.* **426**, 43–55 (2017).
8. R. M. Zayas, et al., The planarian *Schmidtea mediterranea* as a model for epigenetic germ cell specification: analysis of ESTs from the hermaphroditic strain. *Proc. Natl. Acad. Sci. U.S.A.* **102**, 18491–18496 (2005).
9. Y. Wang, J. M. Stary, J. E. Wilhelm, P. A. Newmark, A functional genomic screen in planarians identifies novel regulators of germ cell development. Genes Dev. **24**, 2081–2092 (2010).
10. K. W. Currie, B. J. Pearson, Transcription factors *lhx1/5-1* and *pitx* are required for the maintenance and regeneration of serotonergic neurons in planarians. *Development* **140**, 3577–3588 (2013).
